# Quality of interactions influences everyday life in psychiatric inpatient care—patients’ perspectives

**DOI:** 10.3402/qhw.v11.29897

**Published:** 2016-01-22

**Authors:** Jenny Molin, Ulla H. Graneheim, Britt-Marie Lindgren

**Affiliations:** Department of Nursing, Umeå University, Umeå, Sweden

**Keywords:** Everyday life, experiences, grounded theory, interactions, ordinary relationships, psychiatric inpatient care, patient perspectives, trust

## Abstract

Everyday life consists of daily activities that are taken for granted. It forms the foundation for human efforts and contains elements of both comfort and boredom. Because everyday life escapes no one, life in a psychiatric ward will become ordinary while staying there. This study aims to explore everyday life in psychiatric inpatient care based on patients’ experiences. We individually interviewed 16 participants with experiences of psychiatric inpatient care and analysed the data in accordance with the methods of grounded theory. Data collection and analysis continued in parallel in accordance with the method. Our results showed that everyday life is linked to the core category *quality of interactions influences everyday life*, and three constructed categories—*staff makes the difference*, *looking for shelter in a stigmatizing environment*, and *facing a confusing care content*—were related to the core category. Our results highlight the importance of ordinary relationships between staff and patients in psychiatric inpatient care. These results can be used to develop nursing interventions to improve psychiatric inpatient care and might also be used as a basis for reflective dialogues among staff.

Everyday life consists of daily activities that are taken for granted, and it is synonymous with the ordinary, usual habits; monotony; and routine. Everyday life is the foundation for human efforts and contains elements of both comfort and boredom. Everyday is everyday because it is not linked to the miraculous (Felski, 1999). Because everyday life escapes no one, life in a psychiatric ward will become ordinary while staying there. Lindgren, Aminoff, and Graneheim (2015) showed that the features of everyday life in psychiatric inpatient care imply being surrounded by disorder in a confusing environment where routines and rules are inconsistent but also offer safety. Furthermore, everyday life in a psychiatric ward is characterized by waiting, both “in loneliness” and “in togetherness” (Lindgren et al., 2015). Inpatient care is characterized by patients suffering from various mental disorders, and the medical paradigm predominates among the treatment strategies offered to these patients (Lilja & Hellzén, 2008; Lindgren, Öster, Åström, & Graneheim, 2011; Johansson, Skärsäter, & Danielsson, 2009; Walsh & Boyle, 2009). Research about everyday life in psychiatric inpatient care is sparse, which is unfortunate because providing appropriate inpatient care for people with mental ill-health has been shown to be a complex endeavour (Bowers, 2005).

Research indicates that the ward atmosphere should be supportive and should provide structure and flexibility (Eklund & Hansson, 2001; Johansson, 2006; Middleboe, Schjødt, Byrsting, & Gjerris, 2001) and that the caring culture should offer calmness, security, and personal space (Howard et al., 2001; Johansson et al., 2009; Schröder, Ahlström, & Larsson, 2006). Borge and Fagermoen (2008) showed that a satisfying environment contributed to positive energy and increased the patient's will to live. A recent study reported that locked wards contained contextual factors, such as rules, routines, beds with a belt, and staff handling keys (Lindgren et al., 2015). Furthermore, patients have little space to relax and are not able to protect themselves or to escape. Such factors complicate the care that is provided to the patients. They experience a struggle for worthiness and their days are characterized by waiting for food, medication, and meetings with the staff (Lilja & Hellzén, 2008; Lindgren et al., 2011). When they were not able to receive help from the staff, patients support each other. This is experienced as both helpful and as an emotional burden (Lilja & Hellzén, 2008; Lindgren et al., 2011, 2015; Stenhouse, 2011).

Patients have described psychiatric inpatient care as being locked in one's own lonely world and striving to gain control over one's situation (Lilja & Hellzén, 2008). Furthermore, patients experience a loss of their sense of individuality and are instead seen only as their diagnosis. They experience a lack of human contact, and they feel that professional caregivers prioritize observations and documentation instead of talking to them (Lilja & Hellzén, 2008; Nolan, Bradley, & Brimblecombe, 2011; McAndrew, Chambers, Nolan, Thomas, & Watts, 2014). This is contradictory to patients’ wishes for activities, time for talks, and the ability to have human relations with staff members (Lilja & Hellzén, 2008; Lindgren, Wilstrand, Gilje, & Olofsson, 2004, 2011; Stenhouse, 2011; Walsh & Boyle, 2009). In order for experiences in the ward to be satisfying, research shows that good relationships with staff are needed. Such relationships are characterized by mutual respect, empathy, optimism, trustworthiness, and comfort (Borge & Fagermoen, 2008; Ejneborn Looi, Engström, & Sävenstedt, 2015; Johansson et al., 2009; Nolan et al., 2011; Svensson & Hansson, 2006; Walsh & Boyle, 2009).

In summary, research has focused on descriptions of the psychiatric inpatient care environment, the nurse–patient relationship, and patients’ experiences of care. This study is part of a project aiming to develop an intervention to improve everyday life in psychiatric inpatient care. Such interventions are sparse according to our knowledge. To design successful interventions, it is necessary to have the patients’ point of view. Therefore, the aim for this study was to explore everyday life in psychiatric inpatient care based on patients’ experiences.

## Material and methods

To explore everyday life in psychiatric inpatient care, we used a grounded theory (GT) design (Charmaz, 2014) because we wanted to focus on processes and actions. The method has a constructivist perspective that assumes that there are multiple realities, both processual and constructed. Our perspectives as researchers as well as our interactions with the participants during data collection were taken into account as part of the research reality, and our involvement and subjectivity while interpreting data and constructing categories was central (cf. Charmaz, 2014).

### Context

In Sweden, psychiatric inpatients can be cared for voluntarily as well as involuntarily, in line with Health and Medical Service Act (SFS, 1982:763) or Compulsory Psychiatric Care Act (SFS, 1991:1128). Within an acute psychiatric ward, the patients vary in age, sex, and ethnicity and suffer from various psychiatric disorders. In general, the wards have similar rules and routines regarding locked doors, times for food, access to smoking, possibilities to take a walk, and so on. The wards are staffed by registered nurses, some with and some without specialist training in mental health nursing; enrolled nurses in mental health; a ward manager; and psychiatrists whose time is divided between the wards and other units within the psychiatric clinics.

### Participants

Adults with experiences of psychiatric inpatient care during 2011–2015 from three psychiatric clinics in northern Sweden were recruited from both outpatient and inpatient care as the study progressed. Posters regarding the study were put up on bulletin boards in waiting rooms and public areas at the wards, and persons who wanted to participate were asked to contact the researchers. A total of 16 persons (14 women and 2 men) with experiences from five different wards participated. Their ages ranged from 20 to 51 years (median 31 years). Self-reported diagnoses were borderline personality disorder, depression, bipolar syndrome, posttraumatic stress disorder, eating disorder, dissociative syndrome, anxiety, burnout, and Tourette's syndrome. Four participants reported that their diagnosis was unknown to them. All of the participants had been treated in psychiatric inpatient care between three and seven times, and the patients’ most recent admissions had lasted between a few days and 12 months. Six of the participants were currently admitted to inpatient care at the time of the interview. The most common causes of their most recent admissions were suicidal thoughts or attempts.

### Data collection

Individual interviews were carried out by the first author, but because of personal knowledge of the participants, two interviews were conducted by the last author. The interviews took place either in a room at the clinic or at the university and lasted 39–120 min (median 56 min). The interviews were based on an introductory question: Can you tell me about an ordinary day at the ward? Clarifying questions were added, and in line with the GT method more specific questions about everyday life emerged during the data collection and analysis. The interviews were audio recorded and transcribed verbatim by the first author.

### Analysis

Data collection and analysis continued in parallel to each other, that is, analysis was initiated as soon as the first interview was conducted. By that, questions and ideas from one interview could be deepened and clarified in the next interview. The analysis involved several steps. First, each interview was read through by the authors to get an overall picture of the material. The transcriptions were imported to the Open Code software package (version 4.02) where the initial coding was conducted line by line by the first author. The question “What is happening” and using words that captured actions in data guided the coding. In the focused coding, codes with similar content were grouped and then categories were constructed. For example, codes as *taking initiative, starting to get to know each other, establishing trust, talking about feelings*, and *engaging in helping* were grouped together to form a subcategory. In the theoretical coding, the core category and possible relationships between categories were developed. Constant comparisons were made between codes and between and within the categories, the emerging ideas, and the text. During the process, memos were written and models were drawn. These were used for developing additional questions, and together with discussions in the research group, they were also used as tools for understanding the results. We noticed no additional categories in the material after the ninth interview and at this stage, the core category was defined. Seven more interviews were conducted. No new qualities occurred in the analysis; however, the relations between the categories were clarified.

### Ethical considerations

The Head of the included Clinical Departments of Psychiatry and the Central Ethical Review Board in the region approved the study (Dnr 2014/168-31M), which was performed according to the ethical guidelines described in the Declaration of Helsinki (World Medical Association, 2013). The participants received verbal and written information about the aim of the study, the voluntary nature of participation, their right to withdraw without specifying why, and the confidential nature of the study. All the participants signed an informed consent.

## Results

The results showed that everyday life processes in psychiatric inpatient care are linked to the core category *quality of interactions influences everyday life*. Three constructed categories—*staff makes the difference, looking for shelter in a stigmatizing environment*, and *facing a confusing care content*—were related to the core category. The processes consisted of interactions between different actors, the environment where these interactions took place, and the care content offered in psychiatric inpatient care. The quality of the interactions was what tied these processes together and governed the different parts of everyday life. Having trustful interactions did make up for an otherwise poor environment and a confusing care content whereas adapting to an absence of or to obstructive interactions contributed to a perceived stigmatizing environment and a confusing care content. Each category and its related subcategories will be described below.

### Staff makes the difference

Staffs’ interactions with the participants were central for everyday life processes, and the experiences of these interactions varied among the participants. Subcategories involved in this category were *adapting to absence of interaction with staff*, *adapting to obstructive interactions with staff*, and *having trustful interactions with staff*.

People make the difference … through the way they are, how they treat you, how they look after you, and how they listen to you (P 9)

#### Adapting to absence of interaction with staff

The participants described how they felt invisible to the staff. In such situations, they felt that the staff did not respond to the patients’ questions and instead acted on the basis of rules in such a way that they failed to meet the participants’ needs to express their feelings and talk about daily happenings. They felt that they were not being involved in making decisions about their care and that they sometimes were treated like children even though they were adults. The participants felt that the staff seemed to have difficulties in prioritizing tasks and that they often prioritized activities that did not involve the participants. For example, the staff spent much time in the kitchen and in the laundry room. This was interpreted by the participants as disinterest and a lack of genuine commitment.

The participants described how the staff were often invisible and they often did not know where the staff were. They guessed that they were in the ward office, or as one participant called it, “in the cage”. The office was described as a place where the staff often spent time, and they were difficult to reach when they were there. Either the staff did not open the door when the participants knocked or they did not dare to knock, not wanting to disturb the staff. The participants described how the staff spent time engaging in private matters there, and this was experienced as provocative and made the participants feel unsafe. The staff were often seen playing games on computers and sometimes laughter was heard behind the closed door.

I think it is a big problem that you never see the staff. They sit inside the office all day. You have to stand and knock for a long time if you want to reach them. (P 3)

Sometimes the staff members were unavailable even though they were physically close to the participants. They might walk quickly through the corridor, refer questions to others, or play on their mobile phones while denying the participants assistance or joint activities. This fostered feelings of being ignored and often resulted in patients turning to fellow patients for help. The relationship they lacked with the staff were obtained with each other. These relationships were described as both good and less constructive. It could be nice to socialize with someone with similar experiences, but it could also feel burdensome having to carry someone else's illness when feeling unwell oneself. Some participants described being triggered to self-harm by others’ self-harming behaviours.

#### Adapting to obstructive interactions with staff

The participants described being told by the staff that they took someone else's place who was more deserving of being on the ward, that they did not have to be on the ward, or that they were there too often. They were told that the staff did not know what to do with them and that they were not going to recover. When the participants questioned the staff, they risked being seen as “difficult” and this could lead to being discharged. The participants felt mistrusted, and they felt that they needed to behave well in order to obtain trust and opportunities from the staff. A lack of trust led the participants to feel that they were wasting their time being on the ward. Some participants tested the staff to see if they could be trusted, and this resulted in the perception that only some members of the staff could be trusted.

The participants felt that the staff kept their distance from them by being “too professional”. They saw passivity and a lack of engagement from the staff as equal to poor treatment, and this fostered feelings of being burdensome and a disturbance as well as feelings of anger. The participants experienced that it was the staff against them. To hear the staff making fun of them and talking badly about them was experienced as having their horrors confirmed, a nightmare coming true. Some of the participants adapted to this by avoiding the staff or by pretending to “smile and be happy”, as one participant expressed it.

There were expectations about being treated equally, but also experiences of receiving care related to being liked or disliked as a person. The participants had experiences of staff having favourite patients, and special treatment was seen as wrong. They described receiving less attention if they were quiet and more attention if they acted out. They felt unsafe with some staff members, which fostered feelings of anger and irritation.

They must be able to treat patients equally if they cut themselves or feel bad because it can be very …. I mean, someone like me that already feels that I am totally useless and not worthy to be seen, if staff look after another patient more, then you become even more … I am confirmed that I am not worthy to be seen. (P 10)

The participants experienced that the staff were unable to master their own feelings. When the staff were afraid or insecure and acted on their own feelings, it had consequences for the participants. For example, overreacting because of insecurity and fear silenced the participant. They withheld their thoughts and became careful in sharing their problems so as not to risk losing privileges, for example, a planned furlough. The participants payed close attention to, and were sensitive to, how the staff acted.

The participants experienced being exposed to abuse of power and having to suffer the consequences of the staffs’ behaviour, which was sometimes described as the origin of coercive measures. The participants described how the staff would use unauthorized actions, for example, using beds with a belt without a prescription, how the participants were exposed to reprisals, and hindered from discharging themselves. The descriptions contained accounts of staff being rough, saying mean things, losing their temper, and using force instead of talking. Sometimes, large numbers of staff were used to exert a sense of power over the participants. The participants experienced being threatened by the staff and receiving sermons, and sometimes decisions by the staff were interpreted as punishments. Abuse of power added an external threat to the existing internal threat, as one participant expressed it. It made them feel humiliated, violated, unsafe, and afraid. They described being stripped of their rights and of not having choices. This affected both the current and later contacts with psychiatric care. The exposure to such abusive behaviour was handled by acting carefully and keeping away from the staff so as to avoid any negative consequences. Some of the participants blamed themselves for these abusive incidences, and some blamed the staff. Sometimes they felt like they wanted to beat the staff in self-preservation but the most common defence against such abuse was to abscond from the ward or to discharge oneself too early.

#### Having trustful interactions with staff

The participants saw it as the staff's role to take the initiative to start getting to know the participant if they had not met before. By knowing each other and discussing things together, mutual trust could be established. Building trust also required that the participant see good treatment in action. Trust fostered feelings of being understood, and the participants felt relieved and supported when trust could be established. Such a trusting relationship was necessary for the participants to talk about their feelings, and this contributed to a feeling of safety by knowing that the staff knew how the participant feels.

For me it's very important that the staff recognize me and know who I am and how it has been before, and at the same time see how I am now, that they see that a change has actually occurred … when I feel really bad I might say the same things as before … perhaps there are no other words than the same words, but still you could have come further in some way. (P 6)

The participants described that the staff also cared and engaged in helping the participants. Staff members asked the participants for guidance in how to help them, which was experienced as the staff being engaged and willing to learn. Planning, trying to find solutions together, mutually agreeing on things, and using the participants’ knowledge and wishes made everyday life easier. It was easier to keep agreements if the staff were engaged, and this fostered feelings of being listened to, being involved, having the opportunity to influence care, and having control.

Open dialogue with the staff was described as crucial for everyday life, and one participant expressed that “it was all about the dialogue”. The participants valued dialogue opportunities with physicians and appreciated having the same physician through their whole admission in the ward. Continuous dialogues with the staff facilitated cooperation. If negative decisions needed to be conveyed, explanations were appreciated because these facilitated understanding. It was also valued when the staff were honest about feelings and showed through their actions that the participants were important.

The participants wanted to share responsibility for their care with the staff and expressed that it did not only depend on the staff. Sharing responsibility and being seen as an equal adult made the participants feel more on the same level as the staff. Doing things together and sharing humour was defined as the glue in the relationship, and this brought the participants and staff together and fostered feelings of friendship. Such a relationship made the participants feel that they were working together with the staff, and it facilitated turning to the staff for help and made the participants fond of the staff. This led to carefulness with the relationship; a better understanding of the staffs’ own situation; feelings of hope, motivation to move further; and believing in one's own ability to change.

It is that, to be able to both laugh and at the same time be serious. Because it is when you can get that contact, if you feel that you are on the same level … then you get a different relationship. (P 13)

The participants expressed the importance of being visible to staff, had experiences of being alerted by staff when feeling unwell, and staff taking their time to listen. They saw getting fast support and flexibility as validating. For example, staff making customized exceptions to normal routines made a difference like night and day, as one participant expressed it. It was described by the participants as relieving when the staff recognized them and knew their personal history so that they did not have to explain everything over and over again. For this to be experienced as positive, the staff needed the ability to see and be open for change. Requirements and limitations were also appreciated if staff raised them in a respectful way, and they were experienced as a way for the staff to keep the participant from going adrift.

Small actions were valued by the participants who expressed that they did not ask for much. They had experiences of everyday conversations with the staff, and they stated that small talk provided a nice distraction on the ward. Staff taking their time to sit with the patients was experienced as the staff showing a willingness to help. One participant appreciated the staff sitting at a comfortable distance, whereas another participant valued physical touch. Both actions were interpreted as caring by the participants and made them feel safe.

### Looking for shelter in a stigmatizing environment

The participants’ everyday life took place in an environment where the participants returned for protection but experienced stigma. Their descriptions of the environment were tied to interactions with the staff or the staff's approach to persons with mental ill-health. This category consisted of the subcategories *adapting to a destructive environment* and *searching for safety*.

#### Adapting to a destructive environment

The participants described how being admitted to the ward was like being put in storage. The wards were described as empty of stimuli, unfurnished, and without thought. The staff referred to this as safety, but the participants experienced that comfort was not part of the offer. The staff also, with their words, conveyed that patients should not expect to thrive on the ward. According to the participants, this was experienced as stigmatizing, and they felt that people with mental ill-health had less value as human beings or no rights to exist.

When you get to the ward, there is zero stimuli. There is not a single curtain, and there are only three chairs that are screwed into the wall in the hallway. There are no bedside tables. There is nothing. You only get a feeling that you should not be here. (P 1)

#### Searching for safety

The participants described that being admitted to the ward could be a return to a safe place. They did not want to be there, but they could not remain at home, and the locked door protected them from their behaviour and risks outside the ward. Thus, they were protected against themselves. The participants expressed how recognizing the ward and thriving there could also lead to a sense of being safe, which in turn contributed to recovery.

When I am here, then I know that I cannot harm myself because I'm locked up. It becomes a comfort to me. (P 6)

### Facing a confusing care content

Everyday life entailed a need for opportunities for activity, both spontaneous and planned. In the participants’ descriptions, the content of their everyday lives was connected to, and dependent on, their interactions and relationships with the staff. This category consists of the subcategories *adapting to an unclear structure*, *adapting to passivity*, and *joining in satisfying activities*.

#### Adapting to an unclear structure

The participants experienced an unclear structure where days went by in a blur and routines were inconsistent. They observed that the staff only had time for acute situations, and no one took control. Planned activities were either uncertain or were cancelled, and most of the time nothing happened. Thus, the daily structure of the ward was hard for the participants to understand. The participants wanted to have an overview of the day's schedule and did not want to wait in uncertainty. However, they experienced eternal waiting and described how their days were ruined by not knowing what was going to happen. This created a questioning atmosphere, confusion, and frustration and made it feel like the staff did not care.

One day is very irregular and it differs from day to day. It is very, what should you say … a bit foggy. There is no real knowledge of the patients. (P 7)

The participants often lacked information, and it was often unclear where to turn or whom to talk with. They had needs for, and saw the importance of, daily routines. They were aware that this was necessary for their recovery and to bringing order to their minds. One of the participants described “a need for four walls and someone taking control.”

According to the participants, the unclear structure on the ward actualized the need for leadership by physicians and ward managers. They thought that much depended on the physicians and how they guided the staff, and they expected ward managers to have control over the staff and to influence the ward atmosphere. The participants asked for both physicians and managers to be visible and available for them, especially when problems occurred.

#### Adapting to passivity

The everyday activities on the ward were described as unequal, irregular, and similar to playing the lottery or Russian roulette. Medical calls were irregular, and the staff waited for physicians to make decisions. Thus, the activities planned on the ward were often put on hold. The participants experienced being offered nothing but unfilled time and medication. Sometimes, however, they were offered a chance to play games or cards, to take walks, or have talks with part-time staff.

Most of the time, they socialized with fellow patients, rested, watched TV, or read newspapers. Some opportunities to use exercise equipment, to go to the occupational therapists, and to have daily furloughs were offered, and sometimes schedules were used. The opportunities for spontaneous activities were seen as limited due to the ward's rules. The participants experienced being forced to sacrifice their own needs, and one participant described it like “having a foot chain.” Having to ask staff for permission fostered feelings of not being allowed to do anything.

The participants missed having time for talks and activities, and they experienced that just being locked up on the ward was not helpful. They experienced having to take care of themselves for 24 hours a day, just being locked in at the ward. It was described how staying on the ward could be difficult and boring and how it fostered negative thoughts, hindered recovery, and could increase the desire to self-harm.

You get there, you get a bed, and then … then you do not exist. (P 3)

Most of the participants wanted opportunities to paint, knit, listen to music, exercise, and go outdoors. This could contribute to daily rhythm, provide distractions, help dissipate their stress and anxiety, and could bring the participants hope and empowerment. The participants expressed a need for the staff to be active in offering and joining activities and to sit down for small talks or spontaneous group talks. The staff was expected to be available and to mediate a positive and engaging atmosphere.

#### Joining in satisfying activities

Everyday life was experienced as satisfying by the participants when they could spend time doing things with the staff. The participants believed that this gave the staff more control, allowed the staff to identify the participants’ mood earlier, reduced the feeling of us versus them, and softened the ward climate. Socializing with the staff also created an understanding of the staff and an acceptance of the ward's everyday life situation. Some participants defended the ward and the staff and thought that it was unreasonable to expect the staff to always have time and offer activities. Having such expectations suggested being at the wrong place, as one participant expressed it.

The participants described regular schedules as an advantage and that staff conveyed that daily routines were important. The staff wanted the participants to hold on to and reclaim routines, and they offered to help with this by scheduling activities.

We usually sit down and make a schedule to structure the day because I feel better when I get it structured. (P 4)

Some participants experienced that when the staff tried, they had time for everyone and were good at keeping promises. It was experienced as easy to catch the staff especially when they sat in the corridor socializing with patients and engaging in small talk. The staff's role was described as important, and the participants could see that the staff had plans for them and saw their needs. The participants experienced that the staff knew what to do, had control over the patients’ needs, tried to offer fast help, and arranged for things when asked to.

## Discussion

The aim of this study was to explore everyday life in psychiatric inpatient care based on patients’ experiences. The results showed that the quality of interactions influence everyday life, which consist of processes concerning interactions, environment, and content. Having trustful interactions did make up for an otherwise poor environment and a confusing care content, whereas adapting to an absence of, or to obstructive interactions contributed to a perceived stigmatizing environment and a confusing care content. Unsatisfying interactions with staff fostered anger, which impaired the participant's mood and sometimes led to self-harm. Furthermore, the environment, which has been designed for safety, signalled discomfort and a stigmatizing approach to people with mental ill-health. In contrast, the participants described how a pleasant environment could foster feelings of being safe, which would contribute to fast recovery. And last, the content, with an uncontrolled daily structure and lack of activities, contributed to eternal waiting while daily routines and activities together with staff were needed for recovery. It is noteworthy that care offered in this way is in contrast to the stated aims of being admitted to the ward in the first place.

The findings in the present study showed that all of the participants’ experiences were on the one hand negative and on the other hand positive. It seems that the variation in experiences were general and related to the quality of interaction. Similar results from recent studies reported that staff's interactions with patients have the potential to make a difference in patients’ experiences of everyday life in psychiatric inpatient care. Satisfying interactions were identified as one of the most important aspects of nursing practice (Denhov & Topor, 2011; Wyder, Bland, Blythe, Matarasso, & Crompton, 2015).

In the present study, the participants pointed out that they wanted to spend time with the staff. They valued being familiar with the staff, having dialogues with them, and sharing laughs together. This fostered feelings of friendship. According to Barker and Buchanan-Barker (2007), these kinds of ordinary interactions are often disparaged in the care of psychiatric patients, whereas more refined therapeutic interventions are emphasized. Our results highlight the need for ordinary interactions in everyday life as a central part of the specialized psychiatric care. It is not reasonable to solely offer medical treatment when the patients have needs that require other solutions. Medical treatment needs to be balanced with nursing interventions, and ordinary interactions needs to be emphasized as equally important in treatment. Cleary, Hunt, Horsfall, and Deacon (2012) mean that the staff, through the ordinary, could identify opportunities to interact with patients in meaningful ways. Jackson and Stevenson (2000) showed that the staff need to be able to move between being ordinary and being professional, while still maintaining high levels of empathy. The staff need to establish friendship with the patients by being visible and accessible. Their findings challenge the perception that friendship and closeness with patients could have a negative effect on the patient's treatment and recovery.

According to Barker, Jackson, & Stevenson (1999), some of the most powerful things that nursing staff can do with patients on their recovery journey are imbued with ordinariness. As a part of highlighting the ordinary relations, the staff need to be more aware of the impact of their interactions and how these interactions influence everyday life experiences. Barker and Buchanan-Barker (2007) argue that the staff need to be able to place themselves in the right position and to be a follower rather than a controller. This requires attention to duty, calmness, awareness, and energy. The staff need to not only note what happens to and within the patients but also within themselves. This is echoed by Gunasekara, Pentland, Rodgers, and Patterson (2014) who also highlight the need for attention to the basics of relationships and the importance for the staff to be self-aware. Patient's abilities are strengthened in relation to others, which reinforces the importance of the ordinary relations between the staff and the patients. Through these ordinary relations, the patient can be supported to regain control of their recovery (Ådnøy Eriksen, Arman, Davidson, Sundfør, & Karlsson, 2014; Grant & Briscoe, 2002).

Time is essential when trying to uphold an ordinary relationship. However, it is often described how time is lacking in psychiatric inpatient care. Grant and Briscoe (2002, p. 175) state that “this could be a red herring, since it is more a question of how, rather than how long. A genuine, empathic, respectful interaction with a patient does not need to take longer than a response that lacks these qualities.” The results in the present study showed that time was an issue for the participants. While their time was unfilled, they experienced that staff lacked time even though it was difficult for them to see what the staff did all day. This is echoed both by Shattell, Andes, and Thomas (2008) and Graneheim, Slotte, Säfsten, and Lindgren (2014) who add that nurses’ lack of time hinders the creation of a relationship—the patient will only be a patient, an object that the caregiver needs to care for. One way of dealing with this could be to implement protected engagement time (PET) (Edwards et al., 2008; McCrae, 2014). PET is a fixed period each day during which administrative activities and visiting are suspended so that staff can focus on interactions with patients. It is described as a time to engage for the purpose of strengthening the nurse–patient relationship (Edwards et al., 2008; McCrae, 2014).

Our results showed that the environment was expected to be protective, but it was instead experienced as stigmatizing. Although the protective aspect led to a sense of being safe, which contributed to recovery, the experience of stigma affected the interactions with staff in a negative way. The non-satisfying experiences of the physical environment fostered feelings of not being welcome and of having less value as a human being. This is a barrier for establishing satisfying relationships between the staff and the patients. Shattell et al. (2008) argue that nurses and patients fail to achieve meaningful closeness in the current environment. Similar results were reported by Thibeault, Trudeau, d'Entremont, and Brown (2010) who stated that the environment has an impact on the possibilities for recovery that do not exist without experiences of therapeutic relationships in the context of a comforting physical space. Furthermore, researchers report that, along with the nurse–patient relationship, the ward environment is a main concern in psychiatric inpatient care and that nursing staff are uniquely positioned to shape the environment (Bowers, 2005; Thibeault et al., 2010; Walsh & Boyle, 2009).

The participants in the present study related protection to both the physical environment and to relationships with the staff. Similar results were reported by Muir- Cochrane, Oster, Grotto, Gerace, and Jones (2013) and Johansson et al. (2009) who showed that the ward was a place of refuge that made the patients feel safe and at home. Feeling safe is linked to trust, which is an important aspect of nursing and has been the focus of nursing theory and research. It is essential in the relationship between staff and patients (Gilburt, Rose, & Slade, 2008). Similar results were reported by Pask (1999) who found that trust is central for reducing patients’ anxiety and enabling them to regain a sense of control. Rørtveit et al. (2015) concluded that patients’ experiences of trust are dependent on staff's understanding and commitment in dialogue as well as on meetings being held in a safe environment. They also concluded that feeling safe and being provided with shelter and a homelike environment are associated with a sense of confidence and respect. It is of interest that the participants in our study were told that they should not thrive at the ward. This is in contradiction to what is scientifically known as helpful aspects of recovery.

Staff in mental health services need to embrace the patients’ voices and challenge their own attitudes and preconceptions. Reflective dialogues and peer support can facilitate a more open attitude and contribute to change.

According to our results, daily activities and interactions with the nursing staff were connected to each other in everyday life processes in psychiatric inpatient care. The participants described a need for engaging in activities with the staff, but instead they experienced being left alone with a lack of activities. Lindgren et al. (2015) reported similar results and highlighted the limited time nurses spend with patients and the lack of meaningful activities for the patients being cared for in psychiatric inpatient care. Barker and Buchanan-Barker (2007) also stated that patients in mental health settings experience too little activity and too much enforced passivity.

The participants in the present study experienced that when the staff was busy, activities were the first to be sacrificed in order to ensure time for administrative work and for providing for patient and ward safety (e.g., Cleary & Edwards, 1999; Gunasekara et al., 2014). Kristiansen, Hellzén, and Asplund (2010) found that the nursing staff are most loyal to the practical and task-oriented aspects of their work. Gunasekara et al. (2014) found that making time for interactions in an acute care setting requires the staff to challenge administrative processes and to engage in discussions with colleagues. This requires engagement, empathy, talking with, and listening to patients. Through this, the nursing staff has the opportunity to support patients with mental ill-health on their recovery journeys. Lindgren et al. (2015) suggest that meaningful daily individual or group activities and everyday talks with the patients should be implemented in the daily routines in psychiatric inpatient care.

## Methodological discussion

This study was built on our engagement for people with mental ill-health and how they experience everyday life in psychiatric inpatient care. Because of our experiences as mental health nurses, we have strived to be aware of our preconceptions during data collection (e.g., Dahlgren, Emmelin, & Winkvist, 2007). However, Charmaz (2014) states that if we assume that “social reality is multiple, processual and constructed, then we must take the researcher's position, privileges, perspective and interactions into account as an inherent part of the research reality” (p. 13).

The research process took place in close collaboration between the authors, and the decision about data saturation was discussed carefully. We noted that after the ninth interview, new data did not influence the analysis regarding processes in everyday life. Because this was not clear at once, seven more interviews were conducted. In total, 16 interviews were performed with 14 women and 2 men. One might question the small number of men, but there were only two men who reported interest in participating. This has not been seen as troublesome because we did not aim to investigate whether women and men had different experiences.

Interviewing is a delicate matter, and there might be ethical risks involved in interviewing people with mental ill-health. However, Gaydos (2005) and Biddle et al. (2013) argue that persons are more likely to derive benefit from participating in interview studies than to experience harm. The participants in our study were keen to share their experiences and hoped to contribute to a better everyday life in psychiatric inpatient care. We observed their reactions during the interviews, and signs of discomfort would have led to interruption of the interview and providing help, but this was never the case.

The study was conducted in two county councils and involved three psychiatric clinics and five different wards, which provided variation in the data. This, together with our clinical experiences and a review of the literature, allows us to assume that the results are transferable to other contexts.

These results are not carved in stone. There are possibilities for modification, and further studies where staffs’, physicians’, and managers’ experiences are explored and could contribute to a model for describing processes in everyday life in psychiatric inpatient care.

## Conclusion

It is clear that what the participants in the present study require is neither extravagant nor time consuming or expensive. It is actually quite reasonable and could be timesaving in the end. Quality interactions, that is, closeness to staff in ordinary relationships and spending quality time through simple activities would improve patients’ experiences of everyday life in psychiatric inpatient care and thereby contribute to their recovery. The staff need to review their priorities by reflecting on what kind of activities they engage in. Attention to the simple things can increase the quality of interactions and support the development of an environment that can support recovery. This could be achieved by implementing PET. It is also crucial for staff to have ongoing discussions about their interactions with patients. Regular reflection on their own reactions and actions is also needed, and this could be enabled through reflective dialogues and peer support.

## References

[CIT0001] Ådnøy Eriksen K, Arman M, Davidson L, Sundfør B, Karlsson B (2014). Challenges in relating to mental health professionals: Perspectives of persons with severe mental illness. International Journal of Mental Health Nursing.

[CIT0003] Barker P, Buchanan-Barker P (2007). The Tidal Model. A Guide for Mental Health Professionals.

[CIT0005] Barker P, Jackson S, Stevenson C (1999). The need for psychiatric nursing: Towards a multidimensional theory of caring. Nursing Inquiry.

[CIT0006] Biddle L, Cooper J, Owen-Smith A, Klineberg E, Bennewith O, Hawton K (2013). Qualitative interviewing with vulnerable populations: Individuals’ experiences of participating in suicide and self-harm based research. Journal of Affective Disorders.

[CIT0007] Borge L, Fagermoen M (2008). Patients’ core experiences of hospital treatment: Wholeness and self-worth in time and space. Journal of Mental Health.

[CIT0008] Bowers L (2005). Reasons for admission and their implications for the nature of acute inpatient psychiatric nursing. Journal of Psychiatric and Mental Health Nursing.

[CIT0009] Charmaz K (2014). Constructing grounded theory.

[CIT0004] Cleary M, Edwards C (1999). ‘Something always comes up’: nurse-patient interaction in an acute psychiatric setting. Journal of Psychiatric & Mental Health Nursing.

[CIT0010] Cleary M, Hunt G, Horsfall J, Deacon M (2012). Nurse-patient interaction in acute adult inpatient mental health units: A review and synthesis of qualitative studies. Issues in Mental Health Nursing.

[CIT0011] Dahlgren L, Emmelin M, Winkvist A (2007). Qualitative methodology for international public health.

[CIT0012] Denhov A, Topor A (2011). The components of helping relationships with professionals in psychiatry: Users’ perspective. International Journal of Social Psychiatry.

[CIT0013] Edwards K, Dhoopnarain A, Fellows J, Griffith M, Ferguson D, Moyo L (2008). Evaluating protected time in mental health acute care. Nursing Times.

[CIT0014] Ejneborn Looi G. M, Engström Å, Sävenstedt S (2015). A self-destructive care: Self-reports of people who experienced coercive measures and their suggestions for alternatives. Issues in Mental health Nursing.

[CIT0015] Eklund M, Hansson L (2001). Ward atmosphere, client satisfaction, and client motivation in a psychiatric work rehabilitation unit. Community Mental Health Journal.

[CIT0016] Felski R (1999). The intervention of everyday life. New Formation: A Journal of Culture/Theory/Politics.

[CIT0017] Gaydos H. L (2005). Understanding personal narratives: An approach to practice. Journal of Advanced Nursing.

[CIT0018] Gilburt H, Rose D, Slade M (2008). The importance of relationships in mental health care: A qualitative study of service users’ experiences of psychiatric hospital admission in the UK. BMC Health Services Research.

[CIT0019] Graneheim U. H, Slotte A, Säfsten H. M, Lindgren B-M (2014). Contradictions between ideals and reality: Swedish registered nurses’ experiences of dialogues with in-patients in psychiatric care. Issues of Mental Health Nursing.

[CIT0020] Grant V. J, Briscoe J (2002). Everyday ethics in an acute psychiatric unit. Journal of Medical Ethics.

[CIT0021] Gunasekara I, Pentland T, Rodgers T, Patterson S (2014). What makes an excellent mental health nurse? A pragmatic inquiry initiated and conducted by people with lived experience of service use. International Journal of Mental Health Nursing.

[CIT0022] Howard P. B, Clark J. J, Rayens M. K, Hines-Martin V, Weaver P, Littrell R (2001). Consumer satisfaction with services in a regional psychiatric hospital: A collaborative research project in Kentucky. Archives of Psychiatric Nursing.

[CIT0002] Jackson S, Stevenson C (2000). What do people need psychiatric and mental health nurses for?. Journal of Advanced Nursing.

[CIT0023] Johansson H (2006). Therapeutic Alliance in General Psychiatric Care.

[CIT0024] Johansson I, Skärsäter I, Danielsson E (2009). The meaning of care in a locked acute psychiatric ward: Patients’ experiences. Nordic Journal of Psychiatry.

[CIT0025] Kristiansen L, Hellzén O, Asplund K (2010). Left alone—Swedish nurses’ and mental health workers’ experiences of being care providers in a social psychiatric dwelling context in the post-health-care-restructuring era. A focus-group interview study. Scandinavian Journal of Caring Sciences.

[CIT0026] Lilja L, Hellzén O (2008). Former patients’ experience of psychiatric care: A qualitative investigation. International Journal of Mental Health Nursing.

[CIT0027] Lindgren B-M, Aminoff C, Graneheim U. H (2015). Features of everyday life in psychiatric inpatient care among women who self-harm—An observational study. Issues of Mental Health Nursing.

[CIT0028] Lindgren B-M, Öster I, Åström S, Graneheim U. H (2011). “They don't understand … you cut yourself in order to live.” Interpretative repertoires jointly constructing interactions between adult women who self-harm and professional caregivers. International Journal of Qualitative Studies on Health and Well-Being.

[CIT0029] Lindgren B-M, Wilstrand C, Gilje F, Olofsson B (2004). Struggling for hopefulness: A qualitative study of Swedish women who self-harm. Journal of Psychiatric and Mental Health Nursing.

[CIT0030] McAndrew S, Chambers M, Nolan F, Thomas B, Watts P (2014). Measuring the evidence: Reviewing the literature of the measurements of therapeutic engagement in acute mental health inpatient wards. International Journal of Mental Health Nursing.

[CIT0031] McCrae N (2014). Protected engaged time in mental health inpatient units. Nursing Management.

[CIT0032] Middleboe T, Schjødt T, Byrsting K, Gjerris A (2001). Ward atmosphere in acute psychiatric in-patient care: Patients′ perceptions, ideals and satisfaction. Acta Psychiatrica Scandinavica.

[CIT0033] Muir- Cochrane E, Oster C, Grotto J, Gerace J, Jones J (2013). The inpatient psychiatric unit as both a safe and unsafe place: Implications for absconding. International Journal of Mental Health Nursing.

[CIT0034] Nolan P, Bradley E, Brimblecombe N (2011). Disengaging from acute psychiatric care: A description of users′ experiences and views. Journal of Psychiatric and Mental Health Nursing.

[CIT0035] Open Code 4.02 (2013). ICT Services and System Development and Divison of Epidemiology and Global Health.

[CIT0036] Pask E. J (1999). Trust: An essential component of nursing practice—Implications of nurse education. Nurse Education Today.

[CIT0037] Rørtveit K, Sætre Hansen B, Leiknes I, Joa I, Testad I, Severinsson E (2015). Patients’ experiences of trust in the patient-Nurse Relationship—A systematic review of qualitative studies. Open Journal of Nursing.

[CIT0038] Schröder A, Ahlström G, Larsson B. W (2006). Patients’ perceptions of the concept of the quality of care in the psychiatric setting: A phenomenographic study. Journal of Clinical Nursing.

[CIT0039] SFS 1982:763 Hälso- och Sjukvårdslag.

[CIT0040] SFS 1991:1128 Lag om psykiatrisk tvångsvård.

[CIT0041] Shattell M. M, Andes M, Thomas S. P (2008). How patients and nurses experience the acute care psychiatric environment. Nursing Inquiry.

[CIT0042] Stenhouse R. C (2011). “They all said you could come and speak to us”: Patients’ expectations and experiences of help on an acute psychiatric inpatient ward. Journal of Psychiatric and Mental Health Nursing.

[CIT0044] Svensson B, Hansson L (2006). Satisfaction with mental health services. A user participation approach. Nordic Journal of Psychiatry.

[CIT0045] Thibeault C. A, Trudeau K, d'Entremont M, Brown T (2010). Understanding the milieu experiences of patients on an acute inpatient psychiatric unit. Archives of Psychiatric Nursing.

[CIT0046] Walsh J, Boyle J (2009). Improving acute psychiatric hospital services according to impatient experiences. A user-led piece of research as a means to empowerment. Issues in Mental Health Nursing.

[CIT0047] World Medical Association (2013). Ethical Principles for Medical Research Involving Human Subjects. Declaration of Helsinki.

[CIT0048] Wyder M, Bland R, Blythe A, Matarasso B, Crompton D (2015). Therapeutic relationships and involuntary treatment orders: Service users’ interactions with health-care professionals on the ward. International Journal of Mental Health Nursing.

